# Far infra-red therapy promotes ischemia-induced angiogenesis in diabetic mice and restores high glucose-suppressed endothelial progenitor cell functions

**DOI:** 10.1186/1475-2840-11-99

**Published:** 2012-08-15

**Authors:** Po-Hsun Huang, Jaw-Wen Chen, Chih-Pei Lin, Ying-Hwa Chen, Chao-Hung Wang, Hsin-Bang Leu, Shing-Jong Lin

**Affiliations:** 1 Division of Cardiology, Taipei Veterans General Hospital, Taipei, Taiwan; 2 Department of Medical Research and Education, Taipei Veterans General Hospital, No. 201, Sec. 2, Shih-Pai Road, Taipei, Taiwan; 3 Department of Pathology and Laboratory Medicine, Division of General Laboratory, Taipei Veterans General Hospital, Taipei, Taiwan; 4 Healthcare and Management Center, Taipei Veterans General Hospital, Taipei, Taiwan; 5 Institute of Clinical Medicine, National Yang-Ming University, Taipei, Taiwan; 6 Cardiovascular Research Center, National Yang-Ming University, Taipei, Taiwan; 7 Institute and Department of Pharmacology, National Yang-Ming University, Taipei, Taiwan; 8 Department of Biotechnology and Laboratory Science in Medicine and Institute of Biotechnology in Medicine, National Yang-Ming University, Taipei, Taiwan; 9 Division of Cardiology, Chang Gung Memorial Hospital, Keelung, and Chang Gung University College of Medicine, Taoyuan, Taiwan

**Keywords:** Far infra-red therapy, Endothelial progenitor cell, Diabetes, Ischemia

## Abstract

**Background:**

Far infra-red (IFR) therapy was shown to exert beneficial effects in cardiovascular system, but effects of IFR on endothelial progenitor cell (EPC) and EPC-related vasculogenesis remain unclear. We hypothesized that IFR radiation can restore blood flow recovery in ischemic hindlimb in diabetic mice by enhancement of EPCs functions and homing process.

**Materials and methods:**

Starting at 4 weeks after the onset of diabetes, unilateral hindlimb ischemia was induced in streptozotocine (STZ)-induced diabetic mice, which were divided into control and IFR therapy groups (n = 6 per group). The latter mice were placed in an IFR dry sauna at 34°C for 30 min once per day for 5 weeks.

**Results:**

Doppler perfusion imaging demonstrated that the ischemic limb/normal side blood perfusion ratio in the thermal therapy group was significantly increased beyond that in controls, and significantly greater capillary density was seen in the IFR therapy group. Flow cytometry analysis showed impaired EPCs (Sca-1^+^/Flk-1^+^) mobilization after ischemia surgery in diabetic mice with or without IFR therapy (n = 6 per group). However, as compared to those in the control group, bone marrow-derived EPCs differentiated into endothelial cells defined as GFP^+^/CD31^+^ double-positive cells were significantly increased in ischemic tissue around the vessels in diabetic mice that received IFR radiation. In in-vitro studies, cultured EPCs treated with IFR radiation markedly augmented high glucose-impaired EPC functions, inhibited high glucose-induced EPC senescence and reduced H_2_O_2_ production. Nude mice received human EPCs treated with IFR in high glucose medium showed a significant improvement in blood flow recovery in ischemic limb compared to those without IFR therapy. IFR therapy promoted blood flow recovery and new vessel formation in STZ-induced diabetic mice.

**Conclusions:**

Administration of IFR therapy promoted collateral flow recovery and new vessel formation in STZ-induced diabetic mice, and these beneficial effects may derive from enhancement of EPC functions and homing process.

## Introduction

Angiogenesis, defined as sprouting of blood vessels from preexisting vascular structures, is a physiological response to ischemia but often fails to produce sufficient collateral flow to rescue ischemic organ [1]. In animal models of ischemia, increasing evidence indicates that administration of angiogenic growth factors, either through recombinant protein or by gene transfer, can increase nutrients perfusion through neovascularization and reduce ischemia-related organ damage [2]. However, there is clinical evidence that some patients, unlike healthy experimental animals, fail to develop collateral circulation after tissue ischemia and appear to be refractory to administration of exogenous growth factors [3]. Patients with diabetes are related to endothelial dysfunction and have diminished ability for collateral vessel formation in response to ischemia in the heart and peripheral tissues [4-6].

Convincing evidence suggests that neovascularization in adults is not solely the result of proliferation of local endothelial cells (angiogenesis), but also involves bone marrow-derived circulating endothelial progenitor cells (EPCs) for the processes of vasculogenesis [7]. However, patients with cardiovascular risk factors were shown to have decreased numbers and functions of circulating EPCs [8]. Our recent study also demonstrated that long-term exposure to high glucose may enhance cellular senescence and decrease cell numbers and functional competencies of EPCs via nitric oxide (NO)-related mechanisms [9]. Recent evidence showed that the circulating EPCs levels and arterial stiffness were closely related to their glycemic control in patients with type 2 diabetes mellitus (DM) [10]. These findings provide a rationale for potential therapeutic targets on hyperglycemia-related vascular complications in diabetic patients. Far infrared (IFR) radiation is an invisible electromagnetic wave with a characteristic wavelength between 5.6 and 1000 μm that can be perceived as heat by thermo-receptors in the skin [11,12]. Recent studies indicate that IFR therapy exerts beneficial effects in the cardiovascular system. IFR radiation improves ventricular arrhythmias and endothelial function in patients with heart disease, and enhances access flow and patency of arteriovenous fistulas in hemodialysis patients [13-15]. In addition, IFR therapy promotes microvascular blood flow and angiogenesis in various animal models [16,17]. Although clinical studies have indicated that IFR radiation can exert beneficial effects in the cardiovascular system, the multi-faceted effects of IFR therapy on circulating EPCs and diabetes remain unclear. Therefore, we hypothesized that IFR radiation can improve blood flow recovery after tissue ischemia in diabetic mice and improve functional capacities of EPCs by increasing NO bioavailability.

## Materials and methods

### Animals

Mice at 6–8 weeks old were purchased from the National Laboratory Animal Center, Taiwan (FVB mice). Experimental diabetes was induced in FVB mice by daily intraperitoneal injections of streptozotocin (STZ) in citrate buffer (40 mg/kg) for 5 days for the type 1 diabetic model [18]. Mice were considered diabetic only if they developed glycemia >250 mg/dl and overt glycosuria at 14 days after the first STZ injection. Persistence of diabetes was determined at the end of the study. All mice were kept in microisolator cages on a 12-h day/night cycle. The investigation conforms with the Guide for the Care and Use of Laboratory Animals published by the US National Institutes of Health (NIH Publication 1996). All experimental procedures and protocols involving animals were approved by the institutional animal care committee of National Yang-Ming University (Taipei, Taiwan) and in compliance with the ARRIVE guidelines [19].

### Mouse ischemic hindlimb model

Eight-week-old male wild-type mice and STZ-induced diabetic mice received unilateral hindlimb ischemia surgery by excising the right femoral artery as previously described [20]. Local IFR therapy was given in STZ-induced diabetic mice for 30 min twice per day for 2 weeks after the surgery, and wild-type and diabetic control mice were placed on a heating plate at 34°C for 30 min twice daily to avoid the thermal effect in this study. Briefly, mice were anaesthetized by an intraperitoneal injection of ketamine (100 mg/kg) and xylazine (10 mg/kg). The depth of anesthesia was checked by ensuring that noxious pinch stimulation (blunt forceps) of the hindpaw, the forepaw, and the ear did not evoke any motor reflexes. The proximal and distal portions of the femoral artery were ligated. Hindlimb blood perfusion was measured with a Laser Doppler perfusion imager system (Moor Instruments Limited, Devon, UK) before and after the surgery and followed weekly. To avoid the influence of ambient light and temperature, the results were expressed as the ratio of perfusion in the right (ischemic) versus left (non-ischemic) limb.

### Measurement of capillary density and oxidative stress in the ischemic limb

At 4 weeks after surgery, the mice were euthanized via intravenous ketamine injection. The femora were carefully removed, and the ischemic thigh muscles were embedded in paraffin. Sections (5 μm) were de-paraffinized and incubated with a rat monoclonal antibody against murine CD31 (clone MEC13.1, BD PharMingen, San Diego, CA). Antibody distribution was visualized by using the avidin-biotin-complex technique and Vector Red Chromogenic substrate (Vector Laboratories, Burlingame, CA), followed by counterstaining with hematoxylin. Capillaries were identified by positive staining for CD31. Ten different fields from each tissue preparation were randomly selected, and visible capillaries were counted. Capillary density was expressed as the number of capillaries per square millimeter. To evaluate local oxidative stress levels in ischemic muscles, an antibody against nitrotyrosine (Upstate, Lake Placid, NY, USA) was used.

### Flow cytometry

To investigate the effects of IFR therapy on EPC mobilization in response to tissue ischemia, the fluorescence-activated cell sorting (FACS) Caliber flow cytometer (Becton Dickinson, San Jose, CA, USA) was used to assess EPC mobilization [18]. A volume of 100 μL peripheral blood was incubated with Fluorescein isothiocyanate (FITC) anti-mouse Sca-1 (eBioscience, San Diego, CA, USA), and phycoerythrin (PE) anti-mouse Flk-1 (VEGFR-2, eBioscience) antibodies. Isotype-identical antibodies served as controls (Becton Dickinson, Franklin Lakes, NJ, USA). After incubated for 30 minutes, cells were lysed (PharmLyse; BD Pharmingen), washed with phosphate-buffered saline (PBS), and fixed in 2% paraformaldehyde before analysis. Each analysis included 100,000 events. Circulating EPCs were considered to be from the mononuclear cell population and were gated with double positive for Sca-1 and Flk-1.

### Bone marrow transplantation model

Recipient wild-type mice at 8 weeks of age were lethally irradiated with a total dose of 9.0 Gy [18,20]. eGFP transgenic mice (FVB background) that ubiquitously expressed enhanced GFP (Level Biotechnology Inc., Taipei, Taiwan) were used as the donors. After being irradiated, a recipient mouse received unfractionated bone marrow cells (5×10^6^) from an eGFP mouse by a tail vein injection. Two months after the bone marrow transplantation, the chimeric mice were induced diabetes by daily intravenous injections of STZ as previously described. All mice received a hindlimb ischemic surgery (n = 6 in each group). Repopulation by eGFP-positive bone marrow cells was 95%, as measured by flow cytometry. Two weeks after the induction of hindlimb ischemic surgery in the bone marrow-reconstituted and STZ-induced diabetic mice, tissues were harvested for confocal immunofluorescent and histological analysis. Bone marrow-derived EPCs were stained with antibodies directed against eGFP (Chemicon) and CD31 (BD PharMingen). EPC density was estimated by counting eGFP^+^CD31^+^ double-positive cells (yellow color) under high power field (HPF, ×100) in at least 6 different cross-sections from different animals.

### EPC transplantation in nude mice

Athymic nude mice at 6–8 weeks old were purchased from the National Laboratory Animal Center, Taiwan. Nude mice were then randomly assigned to 5 treatment groups (n = 6 in each group) for intramuscular injection of normal saline, healthy EPC, EPC treated with high glucose for 4 days (EPC-HG), EPC treated with high glucose and IFR for 30 mins (24 hours following by treatment of EPCs in high-glucose conditions for 4 days) (EPC-HG + IFR), and EPC treated with high glucose and transfected with eNOS siRNA (Santa Cruz-Biotechnology Inc., CA, USA) and IFR for 30 mins (EPC-HG + eNOS siRNA + IFR). EPCs were labeled with fluorescent carbocyanine 1,1'-dioctadecyl-1 to 3,3,3',3'-tetramethylindocarbocyanine perchlorate (DiI) dye (Molecular Probes) [21]. Intramuscular injection was performed 24 hours following unilateral hindlimb ischemia srugery. A total volume of 200 μl normal saline or 5 × 10^6^ EPC were injected at 6 sites into the ischemic hind limb distal to the arterial occlusion site. Three ventral injections were placed in the upper limb in proximity to the adductor and semimembranosus muscles. The remaining 3 injections were administered to the ventral lower limb involving the gastrocnemius and flexor digitorum muscles. In order to achieve maximal experimental uniformity, transplanted EPCs were derived from the same donors and used in parallel experiments.

### Human EPC isolation and cultivation

Peripheral blood samples (20 ml) were obtained from healthy young adult volunteers, and total mononuclear cells (MNCs) were isolated by density gradient centrifugation with Histopaque-1077 (1.077 g/ml, Sigma, St. Louis, MO, USA) [20]. Briefly, MNCs (5×10^6^) were plated in 2 ml endothelial growth medium (EGM-2 MV Cambrex, East Rutherford, NJ, USA) with supplements on fibronectin-coated 6-well plates. After 4 days of culturing, the medium was changed and non-adherent cells were removed; attached early EPCs appeared to be elongated with spindle shapes. A certain number of early EPCs were allowed to grow into ECFCs (endothelial colonies forming cells), which emerged 2–4 weeks after the start of the MNC culture. The ECFCs exhibited a cobblestone morphology and monolayer growth pattern typical of mature endothelial cells at confluence [22]. ECFCs were collected and used for all the assays in this study.

### ECFC characterization

The ECFCs were characterized as adherent cells that were positive for endothelial cell and haematopoietic stem cell surface makers, as previously described [20]. The ECFCs were characterized by immunofluorescence staining for the expression of VE-cadherin, platelet/endothelial cell adhesion molecule-1 (PECAM-1, CD-31), and CD34, KDR, AC133 and eNOS (Santa Cruz). The fluorescent images were recorded under a laser scanning confocal microscope.

### Measurement of reactive oxygen species (ROS) production and measurement of nitrate levels

The effect of IFR therapy on ROS production in ECFCs was determined by a fluorometric assay using 2',7'-dichlorofluorescein diacetate (DCFH-DA, Molecular Probes) as a probe for the presence of H_2_O_2_.^21^ ECFCs (10^4^ cells/well) in 96-well plates was incubated in high glucose medium for 4 days. After treatment of IFR radiation, cells were incubated with 20 μmol/L DCFH-DA for 45 minutes. The conditioned medium was measured for nitrate level by Griess reagent [1% sulfanilamide and 0.1% *N*-(1-naphthyl) ethylenediamine in 2% phosphoric acid]. The fluorescence intensity (relative fluorescence units) was assessed at 485-nm excitation and 530-nm emission using a fluorescence microplate reader.

### EPC senescence assay

Cellular aging was determined with a Senescence Cell Staining kit (Sigma). Briefly, after washing with PBS, ECFCs were fixed for 6 minutes in 2% formaldehyde and 0.2% glutaraldehyde in PBS, and then incubated for 12 hours at 37°C without CO_2_ in fresh X-gal staining solution (1 mg/ml X-gal, 5 mM potassium ferricyanide, and 2 mM MgCl_2_; pH6). After staining, green-stained cells and total cells were counted and the percentage of β-galactosidase-positive cells was calculated [20].

### EPC migration assay

The migratory function of ECFCs was evaluated by a modified Boyden chamber assay (Transwell, Costar) [20]. Briefly, isolated ECFCs were detached as described above with trypsin/EDTA, and then 4×10^4^ ECFCs were placed in the upper chambers of 24-well transwell plates with polycarbonate membranes (8-μm pores) that contained serum-free endothelial growth medium. VEGF (50 ng/ml) was added to medium placed in the lower chambers. After incubation for 24 hours, the membrane was washed briefly with PBS and fixed with 4% paraformaldehyde. The upper membrane side was wiped gently with a cotton ball. The membrane was stained using hematoxylin solution and carefully removed. The extent of migration of ECFCs was evaluated by counting the migrated cells in 6 random high-power (×100) microscopic fields.

### Western blotting analysis

ECFCs were lysed in buffer (62.5 mM Tris–HCl, 2% SDS, 10% glycerol, 0.5 mM PMSF, 2 μg/ml aprotinin, pepstatin and leupeptin), and the protein lysates were subjected to SDS-PAGE, followed by electroblotting onto a PVDF membrane [12]. Membranes were probed with monoclonal antibodies against phosphorylated endothelial NO synthase (p-eNOS), eNOS, Akt, p-Akt, VEGF, p-ERK, p-38 MAPK, HO-1 (Cell Signaling) and β-actin (Sigma). Bands were visualized by chemiluminescence detection reagents. Densitometric analysis used ImageQuant (Promega) software.

### Statistical analysis

Results are given as means ± standard errors of the mean (SEM). Statistical analysis was done by unpaired Student’s *t* test or analysis of variance, followed by Scheffe’s multiple-comparison post hoc test. Data were analyzed using SPSS software (version 14; SPSS, Chicago, IL). A p value of < 0.05 was considered statistically significant.

## Results

### IFR therapy promotes blood flow recovery in diabetic mice

**Figure 1:**
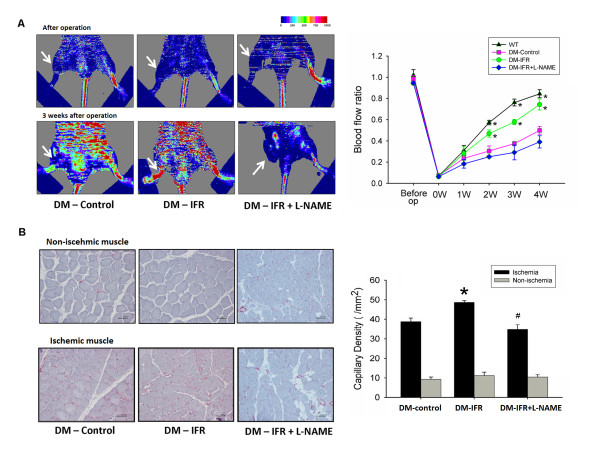
** Effects of far infrared (IFR) therapy on blood flow recovery and new vessels formation in STZ-induced diabetic mice.** (**A**) Representative results of laser Doppler measurements before operation and 4 weeks after hindlimb ischemia surgery in wild-type mice, control (vehicle), IFR therapy, and IFR + N^G^-nitro-L-arginine methyl ester (L-NAME) mice. Color scale illustrates blood flow variations from minimal (dark blue) to maximal (red) values. Arrows indicate ischemic (right) limb after hindlimb ischemia surgery. Doppler perfusion ratio (ischemic/non-ischemic hind limb) over time in the different groups. Administration of L-NAME in drinking water abolished the beneficial effect of IFR therapy in diabetic mice. (*p < 0.05 compared with DM-control; #p < 0.05 compared with DM-FIR; n = 6) (**B**) Mice were sacrificed 3 weeks after surgery and capillaries in the ischemic muscles were visualized by anti-CD31 immunostaining. Results are mean ± standard error of mean (SEM). (*p < 0.05 compared with DM-control; #p < 0.05 compared with DM-IFR; n = 6).

Local IFR therapy was given in STZ-induced diabetic mice for 30 min twice per day for 2 weeks after the surgery, and wild-type and diabetic control mice were placed on a heating plate at 34°C for 30 min twice daily to avoid the thermal effect in this study. As shown in Figure 1A, the STZ-induced diabetic mice without IFR therapy showed delayed blood flow recovery after ischemia surgery compared with that in wild-type mice, as determined by Laser Doppler imaging. Meanwhile, the repeated IFR therapy significantly improved blood flow recovery by 48% in STZ-induced diabetic mice (n = 6 per group). However, the benefit of local IFR radiation was significantly abolished after treatment with the eNOS inhibitor N^G^-nitro-L-arginine methyl ester (L-NAME, 1 mg/ml in drinking water). Consistent with the measurements by Laser Doppler imaging, anti-CD31 immunostaining revealed that repeated FIR radiation increased the number of detectable capillaries in the ischemic muscle in STZ-induced diabetic mice (control versus IFR: 38.8 ± 1.8 versus 48.7 ± 2.4/HPF, p = 0.008) (Figure 1B). However, administration of L-NAME abolished the benefit of IFR radiation (detectable capillaries, IFR versus IFR + L-NAME: 48.7 ± 2.4 versus 34.8 ± 1.7/HPF, p = 0.001).

### Effects of IFR radiation on oxidative stress in ischemic limbs, EPC mobilization and homing process

**Figure 2:**
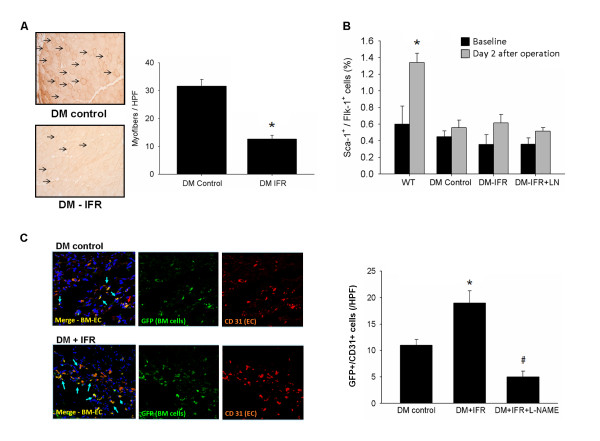
** Effects of IFR radiation on oxidative stress, EPC mobilization after hindlimb ischemia and tissue homing in STZ-induced diabetic mice.** (**A**) Effect of IFR on oxidative stress in ischemic muscles of STZ-induced diabetic mice. Nitrotyrosine (n = 4 per group) immunostaining of ischemic muscles extracted on day 21 in control (vehicle), and in mice that had received IFR radiation. (*p < 0.05 compared with DM-control) (**B**) EPCs (defined as Sca-1^+^/Flk-1^+^ cells) mobilization after tissue ischemia was determined by flow cytometry in STZ-induced diabetic mice given the vehicle, IFR or IFR + L-NAME. (*p < 0.05 compared with WT-baseline; n = 6 per group) (**C**) STZ-induced diabetes was created in FVB mice that received eGFP mouse bone marrow cells. By immunofluorescence staining, STZ-induced diabetic mice in IFR group had more GFP^+^/CD31^+^ double-positive cells in ischemic muscle than those in the vehicle group. (*p < 0.05 compared with DM-control; #p < 0.05 compared with DM-FIR; n = 6).

To further evaluate the effect of IFR therapy on oxidative stress on ischemic muscles, immunostaining against nitrotyrosine was performed. As shown on Figure 2A, assessed by nitrotyrosine staining, a significant reduction of oxidative stress levels in ischemic muscles was noted in diabetic mice that received IFR therapy.

To investigate the effects of repeated IFR radiation on EPC mobilization in response to tissue ischemia, levels of Sca-1^+^/Flk-1^+^ cells in peripheral blood were determined by flow cytometry in STZ-induced diabetic mice (n = 6 per group). EPCs mobilization was enhanced by tissue ischemia in wild-type mice (baseline versus 2 days after operation: 0.60 ± 0.22 versus 1.34 ± 0.11%, p = 0.016). However, impaired mobilization of EPCs in peripheral blood after hindlimb ischemia was noted in STZ-induced diabetic mice (baseline vs. 2 days after operation: 0.45 ± 0.07 vs. 0.56 ± 0.09%, p = 0.374). As shown in Figure 1, administration of local IFR radiation did not increase EPC mobilization in STZ-induced diabetic mice (baseline versus 2 days after operation: 0.36 ± 0.12 versus 0.61 ± 0.10%, p = 0.140) after hindlimb ischemia surgery.

To test the effect of repeated IFR radiation on bone marrow-derived EPC homing and differentiation to endothelial cells, STZ-induced diabetes was created in FVB mice that received eGFP mouse bone marrow cells. By immunofluorescence staining, STZ-induced diabetic mice in the IFR group had more GFP^+^/CD31^+^ double-positive cells in ischemic muscle than those in control group (control versus IFR: 11.2 ± 1.1 versus 18.8 ± 2.0/HPF, p = 0.007, n = 6; Figure 2). In addition, administration of the eNOS inhibitor significantly diminished the effect of IFR radiation on EPC homing. These results suggested that repeated IFR therapy did not promote EPC mobilization after tissue ischemia but may increase circulating EPCs’ homing to ischemic tissue.

### Characterization of Human ECFCs

**Figure 3:**
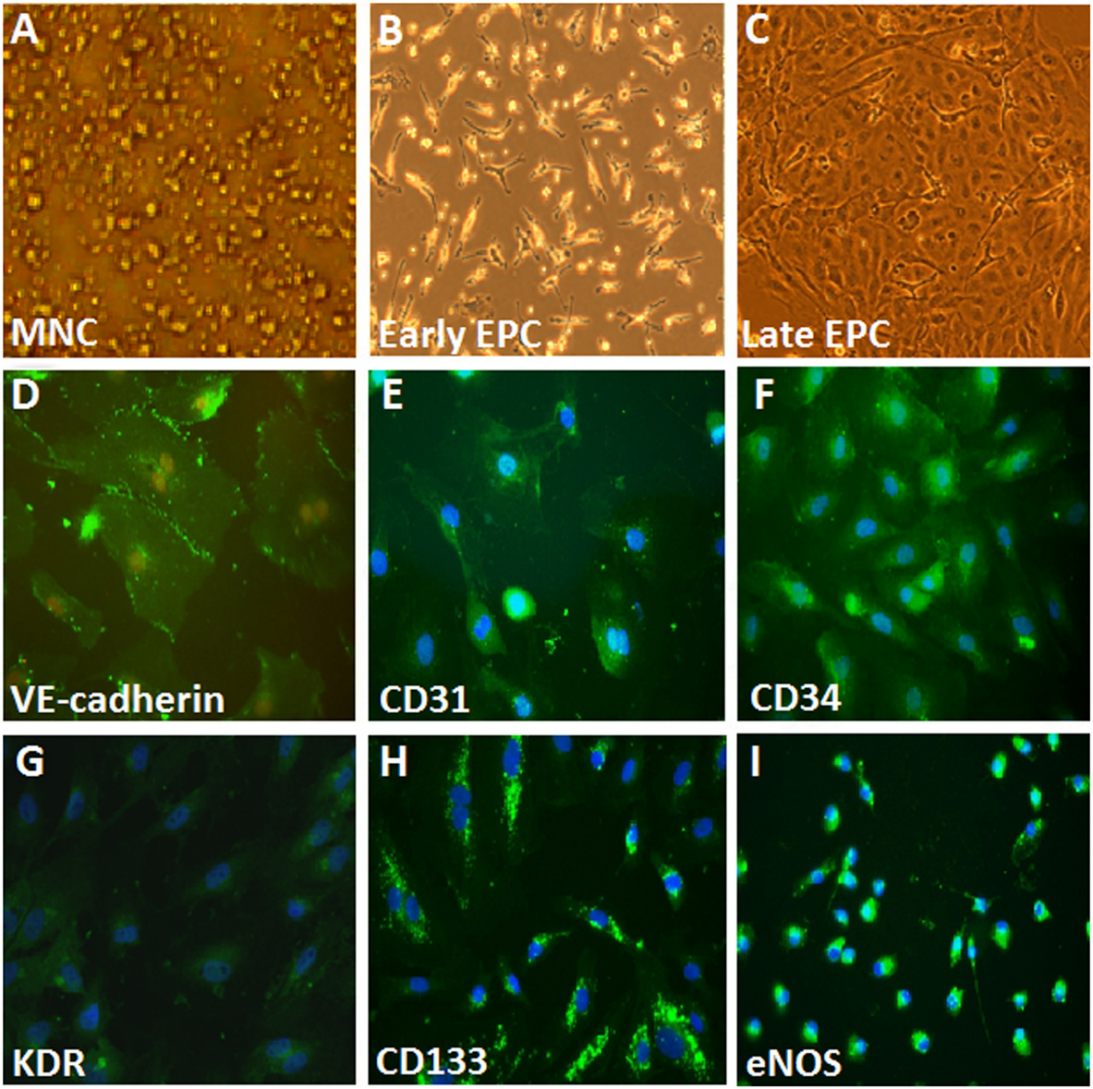
** Morphology and characterization of human endothelial progenitor cells (EPCs) from peripheral blood.** (**A**) Peripheral blood mononuclear cells (MNCs) were plated on a fibronectin-coated culture dish on the first day. (**B**) Four days after plating, adherent early EPCs with a spindle shape were shown. (**C**) Three weeks after plating, ECFCs with a cobblestone-like morphology were selected, reseeded, and grown to confluence. (**D**-**I**) ECFC characterization was performed by immunohistochemical staining. Most of the EPC expressed endothelial and hematopoietic stem cell markers, VE-cadherin, PECAM-1 (CD31), CD34, KDR, AC133, and eNOS, which are considered critical markers of EPCs. Cells were counterstained with 4',6-diamidino-2-phenylindole (DAPI) for the nuclei (blue).

ECFCs were isolated from peripheral blood MNCs of healthy young adult volunteers as previously described [22]. The peripheral blood MNCs that initially seeded on fibronectin-coated wells were in round shape (Figure 3A). After the medium was changed on day 4, attached MNCs appeared to be elongated with a spindle shape (Figure 3B). ECFCs with a cobblestone-like morphology similar to mature endothelial cells were grown to confluence (Figure 3C). ECFCs characterization was performed by immunohistochemical staining, and most of the cells expressed mature endothelial markers, VE-cadherin, PECAM-1 (CD31), CD34, KDR, AC133 and eNOS (Figure 3), which are considered as critical markers of late EPCs. 

### IFR decreases reactive oxidative stress and enhances EPC proliferation, NO production in high glucose conditions

**Figure 4:**
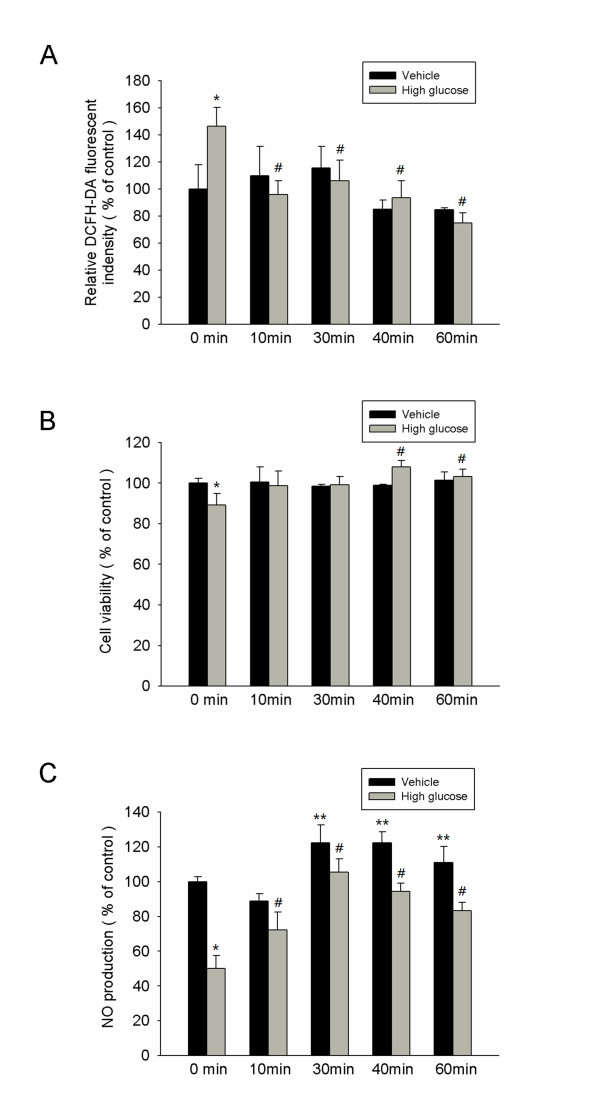
** IFR therapy decreased reactive oxidative stress, recovered EPC proliferation, and increased NO production in high-glucose conditions.** (**A**) High glucose markedly increased H_2_O_2_ production determined by the relative DCFH-DA fluorescent intensity, and the administration of IFR therapy suppressed high glucose-induced reactive oxidative stress (ROS) index in EPCs culture. (*p < 0.05 compared with control - 0 min; #p < 0.05 compared with high glucose - 0 min) (**B**) The effect of IFR radiation on EPCs proliferation was analyzed by MTT assay. (*p < 0.05 compared with control - 0 min; #p < 0.05 compared with high glucose - 0 min) (**C**) Nitrate production (as NO content) in culture medium was measured by Griess reagent. High glucose-suppressed NO production in cultured late EPC s. After 4 days of incubation, IFR radiation increased NO production with or with high glucose conditions. (*p < 0.05 compared with control - 0 min; #p < 0.05 compared with high glucose - 0 min; **p < 0.05 compared with control – 0 min; n = 4 for each experiment).

High glucose markedly increased H_2_O_2_ production determined by the relative DCFH-DA fluorescent intensity, and administration of IFR radiation (10–60 mins) significantly suppressed the high glucose-induced ROS index in cultures of ECFCs (Figure 4A).

As shown in Figure 4B, incubation with high glucose medium for 4 days significantly reduced EPC proliferation (control versus high glucose, 100 ± 2 versus 88 ± 5, p = 0.026). However, administration of IFR radiation for 40 min following by treatment of EPCs in high-glucose conditions significantly reversed the reduction in EPC proliferation in response to high glucose (high glucose versus high glucose + IFR 40 mins, 88 ± 5 versus 109 ± 2, p = 0.006).

High glucose impairs eNOS activation and reduces NO bioavailability in cultured EPCs [7]. We therefore tested the effects of IFR radiation on high glucose-treated EPCs to determine whether IFR could recover impaired NO production in EPCs. After 4 days of incubation in 25 mM high glucose medium, the NO production in cultured medium was significantly decreased (Figure 4C). However, administration of IFR radiation for 30 mins following by treatment of EPCs in high-glucose conditions can significantly upregulate high glucose-impaired No production.

### IFR radiation upregulates phosphorylation of eNOS, and enhances VEGF in high glucose conditions

**Figure 5:**
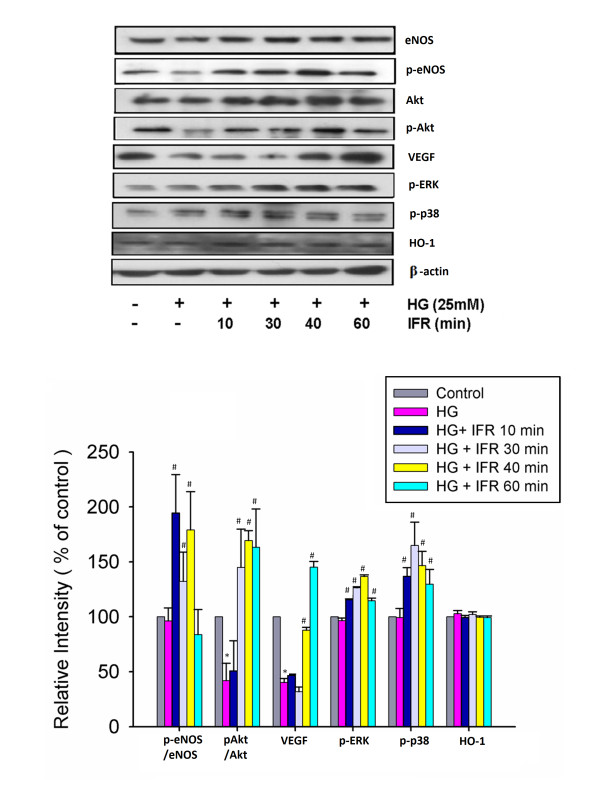
** Effects of IFR radiation on eNOS, Akt, p-ERK, p-38 MAPK, VEGF and HO-1 production in cultured EPCs.** Administration of IFR radiation on cultured ECFCs for 10, 30, and 40 min followed by treatment of EPCs in high-glucose conditions significantly upregulated high glucose-impaired eNOS production and eNOS activity (p-eNOS/total eNOS). Treatment of IFR therapy for 30, 40 and 60 min also upregulated Akt activation (p-Akt/total Akt). In addition, administration IFR radiation also promoted VEGF production, p-ERK, and p-38 MAPK in high glucose conditions, but not HO-1. Each bar graph shows the summarized data from four separate experiments by densitometry after normalization. Data are means ± SEM; n = 4 in each experiment. (*p < 0.05 compared with control; ^**#**^p < 0.05 compared with HG group.).

We investigated the effects of IFR radiation on high glucose-treated EPCs to determine whether IFR therapy could recover impaired eNOS activation in EPCs. Administration of IFR radiation on cultured ECFCs for 10, 30, and 40 min followed by treatment of EPCs in high glucose conditions significantly upregulated high glucose-impaired eNOS production and eNOS activity (p-eNOS/total eNOS). Treatment of IFR therapy for 30, 40 and 60 min also upregulated Akt activation (p-Akt/total Akt). In addition, administration IFR radiation also promoted VEGF production, p-ERK, and p-38 MAPK in high glucose conditions, but not HO-1 (Figure 5). These findings suggested that treatment with IFR radiation may enhance activities of eNOS, Akt, p-ERK, p-38 MAPK and VEGF production in EPCs in response to high glucose stimulation.

### IFR improves high glucose-suppressed EPC functions and senescence in vitro

**Figure 6:**
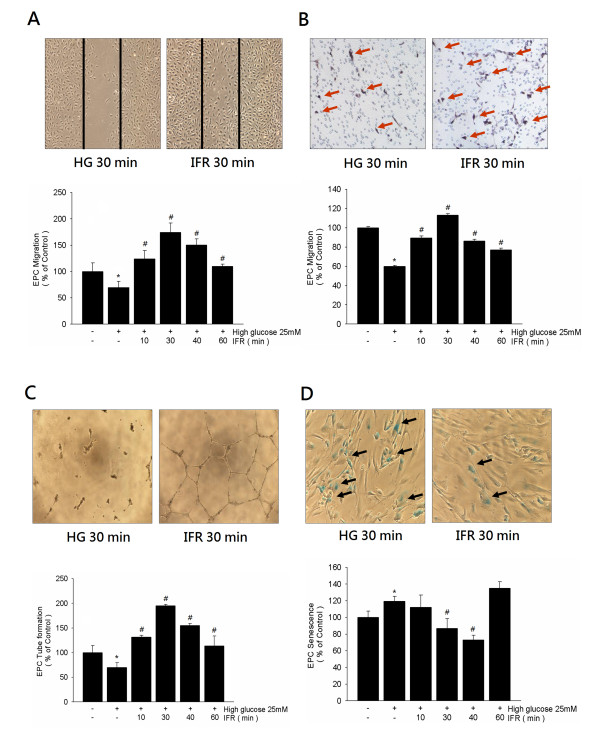
** Effects of IFR therapy on EPC migration, tube formation, and senescence in vitro.** (**A**,**B**) Scratch test and modified Boyden chamber assay were used to assess the migratory function of ECFCs in high glucose conditions. Boyden chamber assay using VEGF as a chemoattracting factor was used to evaluate the effects of IFR radiation on EPC migration. (**C**) An in vitro angiogenesis assay for late ECFCs used ECMatrix gel. Representative photos for in vitro angiogenesis are shown. Cells were stained with crystal violet, and the averages of the total area of complete tubes formed by cells were compared by using computer software. (**D**) To determine the onset of cellular aging, acidic ß-galactosidase was used as a biochemical marker for acidification, typical for ECFCs senescence. Data are means ± SEM; n = 4 in each experiment.

To investigate the effects of IFR radiation on EPCs, we used both scratch test and modified Boyden chamber assay to assess the migratory function of EPCs in high glucose conditions. Compared with the control group, incubation of EPCs with high glucose (4 days) significantly decreased EPC migration (100 ± 9 versus 63 ± 11/HPF, p = 0.031). However, treatment with IFR radiation for 30 minutes significantly recovered high glucose-suppressed late EPC migratory function (high glucose versus high glucose + IFR, 63 ± 11 versus 174 ± 10 cells/HPF, p = 0.005; Figure 6A). Similar findings were observed by modified Boyden chamber assay (Figure 6B).

After 4 days of culturing, the capacity for tube formation of EPCs on ECMatrix gel was significantly reduced in the presence of high glucose compared with the control group, whereas treatment with IFR radiation for 30 mins followed by treatment of EPCs in high-glucose conditions ameliorated this high glucose suppressed-tube formation by EPCs (100 ± 15 vs. 195 ± 3 cells/HPF, p = 0.003; Figure 6C).

Compared with the control group, incubation of EPCs with high glucose significantly increased the percentage of senescence-associated ß-galactosidase-positive EPCs (100 ± 6 versus 119 ± 5%, p = 0.032). Administration of IFR radiation (30 mins) followed by treatment of EPCs in high-glucose medium significantly attenuated the percentage of senescence-associated ß-galactosidase-positive EPCs (high glucose versus high glucose + IFR, 119 ± 5 versus 87 ± 9%, p = 0.013; Figure 6D).

### EPC treated IFR radiation transplantation improves hindlimb perfusion

**Figure 7:**
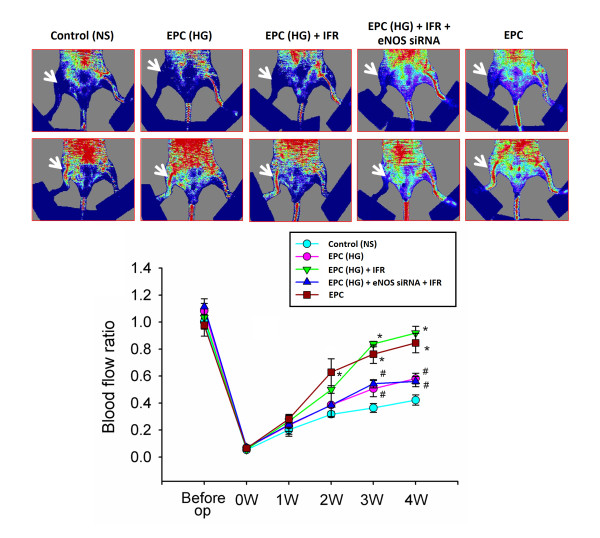
** IFR treated-EPC transplantation improved blood perfusion in the ischemic hindlimb.** (A) Representative images of hindlimb blood flow measured by laser Doppler and quantitative analysis of blood flow expressed as perfusion ratio of the ischemic to the contralateral (non-operated) hindlimb immediately after hindlimb ischemia surgery and 3 weeks after intramuscular injection of normal saline, EPC-treated with high glucose (EPC-HG), EPC-treated with high glucose and FIR therapy (EPC-HG + IFR), or EPC treated with high glucose and IFR and eNOS siRNA (EPC-HG + eNOS siRNA + IFR). (*p < 0.05 compared with control; #p < 0.05 compared with EPC-HG + IFR; n = 6).

In the animals receiving normal saline, blood flow remained constant throughout the study, around 42% of that measured in the non-ischemic limb (42 ± 8%, four weeks after operation). In contrast, the mice transplanted with EPC and EPC received IFR therapy (30 mins) in high glucose medium (EPC-HG + IFR), but not EPC in high glucose medium without IFR therapy (EPC-HG) and EPC treated with high glucose and IFR and eNOS siRNA (EPC-HG + eNOS siRNA + IFR) already showed a significant improvement in blood flow by three weeks after EPCs implantation (both p < 0.05; Figure 7). Consistent with the measurements by Laser Doppler imaging, anti-CD31 immunostaining revealed that transplantation of EPC and EPC treated with IFR in high glucose conditions significantly increased the number of detectable capillaries in the ischemic muscle than mice received normal saline (both p < 0.05 compared to normal saline). However, treatment with eNOS siRNA abolished the benefit of IFR radiation in high glucose medium (IFR versus IFR + eNOS siRNA: 38.7 ± 4.7 versus 21.3 ± 3.7/HPF, p = 0.015, n = 6 per group).

## Discussion

This study first to show a favorable effect of IFR radiation on ischemia-induced neovascularization in diabetic mice. Repeated IFR radiation enhanced blood flow recovery and new vessel formation in ischemic hindlimbs, and the beneficial effect may be derived from enhancement of EPC homing process by reduction of oxidative stress in ischemic tissue rather than promotion of EPC mobilization. In addition, direct IFR radiation could ameliorate high glucose-induced oxidative stress, attenuate cellular senescence and improve EPC functions. Mice received EPC treated with IFR radiation showed a significant improvement in blood flow recovery after tissue ischemia in comparison to those received normal saline. Given the evidence mentioned above, our findings indicated the direct beneficial effects of IFR radiation on blood flow recovery after tissue ischemia, and IFR radiation counteracts the detrimental effect of a diabetic environment on improvement of EPC functions, which may provide some novel rationales for its potential clinical impact on vascular protection.

Improved neovascularization in response to tissue ischemia is an important therapeutic strategy to reduce organ damage. Convincing evidence suggests that neovascularization in adults is not solely the result of the proliferation of endothelial cells (angiogenesis) but also involves circulating EPCs in the process of vasculogenesis [7]. These circulating EPCs are derived from bone marrow and are mobilized endogenously, triggered by tissue ischemia, or exogenously by cytokine stimulation, such as VEGF and stromal cell-derived factor-1 (SDF-1) [23].

However, patients with diabetes or cardiovascular risk factors were shown to have decreased numbers and function of circulating EPCs [8,24,25]. Recent studies indicated that advanced glycation end products (AGEs) promoted EPC apoptosis [26], and long-term exposure to high glucose may enhance cellular senescence and reduce cell numbers and functional competencies of EPCs via NO-related mechanisms [10]. Mobilization and differentiation of EPCs are modified by NO, and bone marrow-expressed eNOS is essential for the mobilization of stem and progenitor cells [27]. In addition, endogenous NOS inhibitors, such as asymmetric dimethylarginine (ADMA), were shown to suppress EPC differentiation and function, and contributed to impaired endothelial function [28]. These findings provide a rationale for potential therapeutic targets for hyperglycemia-suppressed EPC functions, and diabetes-related vascular complications.

Diabetic patients frequently suffer from micro- or macrovascular abnormalities, including retinopathy, nephropathy, neuropathy and accelerated atherosclerosis. It is evident that decreased bioavailability of NO produced from eNOS plays a crucial role in the development and progression of atherosclerosis. Under various pathological conditions such as type 2 diabetes eNOS may become dysfunctional or its expression may be decreased. Endothelial dysfunction is associated with childhood obesity and is closely linked to the amount and function of EPCs, and a combined after-school exercise program was shown to increase circulating EPC levels by enhancement of NO bioavailability [29]. Moreover, enhancement of oxidative stress by tissue ischemia may downregulate NO bioavailability because free radicals can directly inactivate NO [30]. Inadequate angiogenic response to ischemia in the ischemic limbs or myocardium of diabetic patients could result in poor collateral formation and severe organ damage.

IFR radiation is an invisible electromagnetic wave with a characteristic wavelength between 5.6 and 1000 μm that can be perceived as heat by thermo-receptors in skin [11,12]. The technology of IFR has been applied widely in a variety of fields. The thermal effect of IFR results in vasodilation and increasing tissue blood flow. Local IFR therapy may allow multiple energy transfer as deep as 2 to 3 cm into subcutaneous tissue without irritating or overheating the skin like unfiltered heat radiation [31]. The skin temperature steadily increased to a plateau at approximately 38 to 39°C during the treatment of FIR for 30 to 60 min as long as the distance between the ceramic plate and the skin was >20 cm [16]. Therefore, infrared therapy can be free of the disadvantages or adverse effects of thermal therapy. In this study, wild-type and diabetic control mice were placed on a heating plate at 34°C for 30 min twice daily to avoid the thermal effect between groups. The rectal temperature was also assessed in these mice, and showed no temperature elevation in mice received IFR therapy compared to those without IFR radiation.

In addition to the thermal effect, increasing evidence suggests nonthermal effects of IFR therapy exert beneficial effects in the cardiovascular system through NO-related pathway [31,32]. In animal studies, Akasaki and colleagues demonstrated that repeated IFR therapy could upregulate eNOS expression and augment angiogenesis in an apolipoprotein E–deficient mouse model of unilateral hindlimb ischemia [16]. In human study, Imamura et al. showed that two weeks of repeated sauna therapy significantly improved vascular endothelial function, resulting in an increase of flow-mediated, endothelium-dependent dilation of the brachial artery from 4 to 5.8% in patients with coronary risk factors [14]. These findings suggested nonthermal effect of IFR may derive from upregulation of eNOS activity and enhancement of NO bioavailability. In the study, we demonstrated that repeated IFR therapy could activate eNOS and Akt, and upregulate the migration and tube formation capacities of ECFCs. Akt is downstream from PI 3-kinase and is capable of directly phosphorylating eNOS at Ser^1179^, resulting in its activation. Recent evidence also indicated that p-38 MAPK plays a key role in downregulating EPCs by hyperglycemia in diabetic patients [33]. EPCs exposed to IFR upregulated expression of p-38 MAPK and p-ERK. These findings are in line with recent study showing that IFR radiation significantly promoted angiogenesis by a MAP kinase dependent pathway on mature endothelial cells. Mice received EPC treated with IFR radiation in high glucose conditions showed a significant improvement in blood flow recovery after tissue ischemia compared with those received normal saline. These beneficial effects may provide some novel rationale for the vascular protective properties of IFR therapy in diabetic patients with critical limb ischemia or clinical implication to treat dysfunctional EPCs before cell therapy.

## Conclusions

This study provides the first evidence that the administration of IFR therapy promoted collateral flow recovery and new vessel formation in STZ-induced diabetic mice. These beneficial effects may derive from enhancement of EPC functions and homing process, which provide some novel rationale for the vascular protective properties of IFR therapy in diabetic patients with critical limb ischemia or further clinical implication in cell therapy.

## Abbreviations

EPCs: Endothelial progenitor cells; NO: Nitric oxide; DM: Diabetes mellitus; IFR: Far infrared; STZ: Streptozotocin; FACS: Fluorescence-activated cell sorting; FITC: Fluorescein isothiocyanate; PE: Phycoerythrin; PBS: Phosphate-buffered saline; MNCs: Mononuclear cells; ECFCs: Endothelial colonies forming cells; PECAM-1: Platelet/endothelial cell adhesion molecule-1; ROS: Reactive oxygen species; DCFH-DA: 2',7'-dichlorofluorescein diacetate; p-eNOS: Phosphorylated endothelial NO synthase; L-NAME: N^G^-nitro-L-arginine methyl ester; SDF-1: Stromal cell-derived factor-1; AGEs: Advanced glycation end products; ADMA: Asymmetric dimethylarginine.

## Competing interests

The authors declare that they have no competing interests.

## Authors’ contributions

Po-Hsun Huang conducted the experiments and contributed to the study implementation, statistical analysis, interpretation, and the preparation of the manuscript. Jaw-Wen Chen contributed to the study conception and design, and the preparation of the manuscript. Chih-Pei Lin, Ying-Hwa Chen, Chao-Hung Wang, and Hsin-Bang Leu helped to conduct the experiments and contributed to the study conception and design, implementation, and interpretation. Jaw-Wen Chen and Shing-Jong Lin supervised the study conduction and contributed to the study conception and design, implementation, statistical interpretation, the preparation and finalization of the manuscript. All authors approved the final manuscript for publication.
